# Thermal conductivity of graphene coated copper under uniaxial tensile mechanical strain†

**DOI:** 10.1039/d5na00088b

**Published:** 2025-05-08

**Authors:** Micah P. Vallin, Hisato Yamaguchi, Rijan Karkee, Chanho Lee, Ramon M. Martinez, Saryu J. Fensin, Jun Beom Park, Hi Tin Vo, Richard Z. Zhang, Michael T. Pettes

**Affiliations:** a Center for Integrated Nanotechnologies, Materials Physics and Applications Division, Los Alamos National Laboratory Los Alamos NM 87545 USA pettesmt@lanl.gov; b Department of Mechanical Engineering, University of North Texas Denton TX 76207 USA zihao.zhang@unt.edu; c Applied Electrodynamics Group, Accelerator Operations and Technology Division, Los Alamos National Laboratory Los Alamos NM 87545 USA; d Materials Science in Radiation and Dynamics Extremes (MST-8), Materials Science and Technology Division, Los Alamos National Laboratory Los Alamos NM 87545 USA

## Abstract

Graphene continues to demonstrate promise as a highly effective barrier coating, even at only one atom thick. The thermal properties of this coating are also promising to allow diffusion of heat across the surface, as the isolated graphene is an intrinsically good thermal conductor. However, this and its behavior under mechanical deformation have been less extensively studied. This report demonstrates that the in-plane thermal conductivity and interfacial thermal conductance of graphene coatings on copper are affected by mechanical strain. By inducing strain in the copper substrate, the Raman-active 2D peak exhibits a change in position and a change in laser power dependence as the copper substrate is uniaxially elongated to a maximum of 0.5%. Non-linear trends in thermal conductivity are observed with tensile strain in samples with differing strain transfer rates from the substrate, indicating the close correlation between intrinsic thermal conduction and interfacial properties in atomically thin coatings transferred onto metals.

## Abstract 2

Advanced graphene coatings perform well under conditions where mechanical deformation is expected. We demonstrate that the in-plane thermal conductivity of a graphene coating drops when undergoing even very small uniaxial tensile deformations.

## Graphical abstract

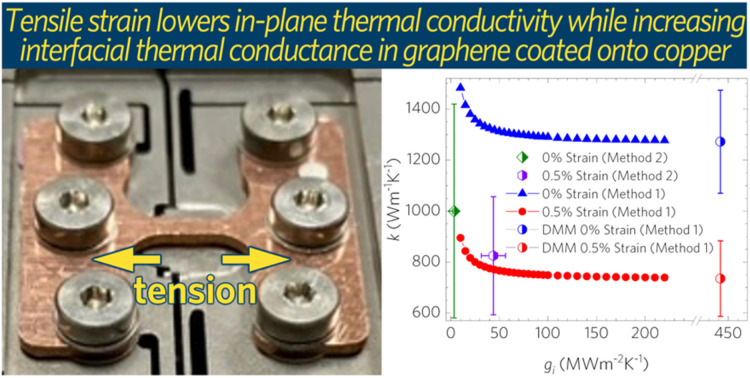

## Introduction

Graphene has received extensive attention and research due to its promise in applications across various technological areas, including as a highly effective barrier coating.^1–4^ Thermal transport of the barrier coating is also of interest as it affects heat dissipation; graphene has high in-plane thermal conductivity at room temperature, ranging from 1500–1800 W m^−1^ K^−1^ to as high as ∼5300 W m^−1^ K^−1^ (ref. 5–8) for suspended samples and ∼500–600 W m^−1^ K^−1^ when it comes in contact with polymer^9^ or silicon dioxide.^10^ While studies on the thermal conductivity of carbon-based materials are numerous, one effect which has recently begun to receive attention is that of strain. Recently, suspended carbon nanotubes were shown to exhibit a doubling of thermal conductivity with only 0.39% uniaxial tensile strain.^11^ In graphene, experimental observations have shown that the Raman peak shifts experience changes due to strain,^12–14^ although the effects of strain on thermal conduction have primarily been reported through first principles and modeling investigations.^15,16^ The calculations performed by Kuang *et al.*^15^ and Pereira *et al.*^16^ both indicate that the phonon dispersion of graphene shifts to a lower frequency with increasing tensile strain; however, Kuang *et al.*^15^ reported that the thermal conductivity may diverge with respect to the reciprocal space mesh sampling size when strain is applied to the crystal structure. Similarly, the thermal conductivity values reported by Pereira *et al.*^16^ diverge with respect to time when strain is applied; however, the thermal conductivity reaches a converged value for 1% strain at temperatures of 300 K and 800 K. The thermal conductivity was predicted to be higher for the 1% strain case than for the unstrained case.^16^ Experimentally, graphene has recently been shown to exhibit a decrease in thermal conductivity with strain when placed on a flexible polydimethylsiloxane substrate.^17^

Experimentally, thermal conductivity can be measured in many ways with varying degrees of difficulty and uncertainty. One method developed as a non-contact diagnostic, referred to generally as the optothermal Raman technique, is a useful non-destructive probe for measuring thermal conductivity and interfacial transport properties of two-dimensional (2D) materials,^7,8,17–23^ making it a versatile method for measuring the thermal conductivity of a variety of 2D coatings.

Here, we report the thermal conductivity of monolayer graphene coated onto a copper substrate before and after annealing, using the optothermal Raman technique. We demonstrate the effect that straining the substrate into the plastic regime has on the effective thermal conductivity of the graphene coating, which is considerable even in the case of weak strain transfer.

## Experimental

**Fig. 1:**
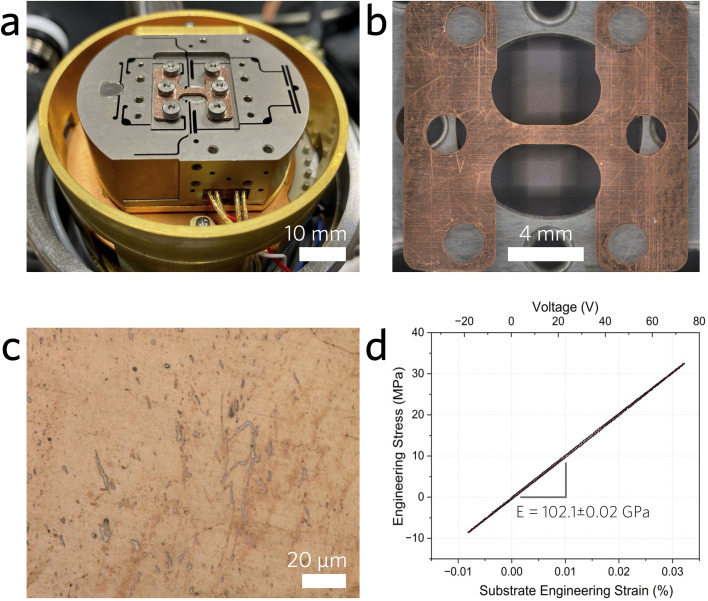
(a) Razorbill UC-200 stress–strain cell with a graphene-coated Cu dog bone substrate assembled for strain experiments. (b) Low- and (c) high-resolution optical micrographs of the graphene-coated Cu dog bone. (d) Representative stress–strain curve taken to validate the engineering strain in the Cu substrate.

Monolayer graphene was synthesized by chemical vapor deposition^24^ and wet-transferred^25^ to a polished copper (Cu) machined dog bone substrate. The Cu dog bones were ground using standard SiC paper of varying grit (P800#, P1200#, P2400#, and P4000#), and subsequently polished using water-based alumina slurry (1.0 and 0.3 μm size) on a Nap polishing cloth. Raman measurements were conducted in the center of the Cu dog bone gage area, where the strain effects were localized by design^26^ (see the ESI†). The thickness of graphene was additionally confirmed from atomic force microscopy on SiO_2_/Si substrates.^27^ The graphene was annealed on the Cu substrates for 2 weeks at 475 K and ∼10^−6^ mbar in order to ensure optimal contact between the graphene sample and the Cu substrate. Images of graphene-coated Cu dog bones used in our experiments can be found in Fig. 1.

Raman spectroscopic measurements were performed in reflection mode using 532.3 nm continuous wave excitation (Oxxius LCX-532S-100, CW single longitudinal mode diode pumped solid state laser) on a Horiba LabRAM HR Evolution high resolution confocal Raman microscope. The experiment was configured using an 1800 mm^−1^ holographic grating blazed at 500 nm, a 350 μm confocal hole diameter, and either a 20×, 0.45 NA or a 50×, 0.7 NA glass-corrected semi-apochromat objective (LCPLFLN20XLCD, LCPLFLN50XLCD, Olympus). Spectral calibration was performed using the 1332.5 cm^−1^ band^28^ of a synthetic Type IIa diamond, and spectral intensity was calibrated using a VIS-halogen light source (NIST test no. 685/289682-17).

Uniaxial tensile strain was applied using a stress–strain cell (UC-200, Razorbill Instruments) inside of a variable temperature optical cryostat (MicrostatHiRes, Oxford Instruments). The engineering strain in the Cu substrate is denoted as *ε*_substrate_, and it cannot be assumed that the entirety of this strain is uniformly imparted to the graphene coating due to local strain inhomogeneity that may induce slippage and alter the conformality of the coating.^29^ Discrepancies in modulus can arise mainly from instrumental factors such as sample mount stiffness, which is the dominant source of error in the displacement. Resetting the strain cell to a reproducible zero position before each test and introducing a small dwell (0.1 second) at every voltage step ramp improved the measured elastic modulus to 102.1 GPa and reduced hysteresis (Fig. 1d). The hysteresis curve arises from a combination of deformation of the gage area and any sample mount slippage, which is unlikely given the narrow copper sample and wide mounting area. Finite element modeling is shown in Fig. S1.† Additionally, stress–strain behavior in the plastic deformation regime is shown in Fig. S2,† where hysteresis can be seen as the amount of work performed on the sample.

To accurately measure the Raman peak shifts with respect to temperature, Raman spectra were recorded with a 50×, 0.7 NA objective beginning at the annealing temperature of 475 K and decreasing to 300 K in increments of ∼15 K. Similarly, incident power dependent Raman measurements were chosen such that they would provide a wide range of laser powers as well as provide a clear range of wavenumber peak shifts, 0.7–25.3 mW, measured using a lock-in power meter (RM9, RM1C chopper, and EA-1, Ophir-Spiricon, LLC). From these power dependent and temperature dependent peak shift coefficients, *χ*_P_ and *χ*_*T*_, respectively, were used in the extraction of thermal conductivity, *k*, in a one-microscope objective technique (denoted as method 1), and both *k* and interfacial conductance, *g*_i_, in a two-objective technique (denoted as method 2) (see the ESI†).

**Fig. 2:**
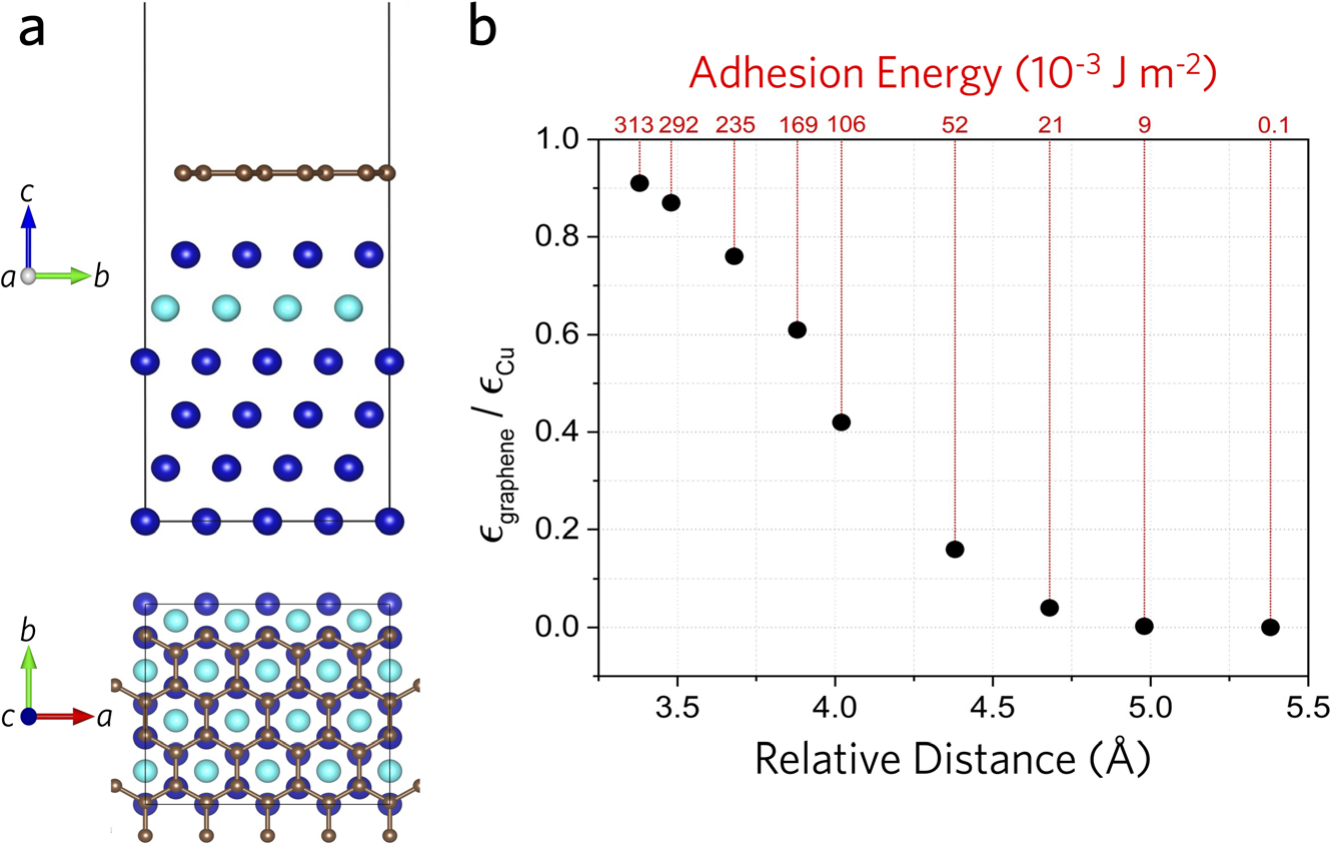
(a) Side (upper) and top (lower) views of Cu(111)/graphene top-fcc stacking. Cu atoms are shown in blue, with the penultimate Cu layer rendered in turquoise to highlight the top-fcc configuration; C atoms are grey. (b) Computed strain transfer from the Cu substrate to the single-layer graphene coating as a function of the graphene–Cu separation distance. Red values above the top axis give the corresponding adhesion energy.

Density functional theory (DFT) calculations were performed with the plane-wave approach implemented in the QUANTUM ESPRESSO^30^ software. The local density approximation (LDA) functional was selected because it provides lattice parameters and properties close to experimental values. Typically, semi-empirical van der Waals corrections (such as Grimme-D2) are used for layered materials and interfaces. However, since our adhesion energy calculations involve metallic surfaces [Cu(111)], which are not well described by van der Waals corrections,^31,32^ we employed only the LDA functional without these corrections. We used a kinetic energy cutoff of 1088 eV and a half-shifted *k*-point grid of 10 × 10 × 1. The copper slab was modeled using the Cu(111) surface, the most common and stable surface of copper with minimal structural changes upon formation.^33^ Previous studies on Cu–graphene interfaces indicated that five copper layers are sufficient to achieve convergence;^34^ hence, we chose a six-layer copper slab for greater accuracy. Following the approach of Shi *et al.*,^34^ we fixed the two bottom layers during structural relaxation. A 2 × 2 supercell was constructed (Fig. 2a), containing four copper atoms per layer and eight graphene atoms per supercell, resulting in a lattice mismatch of 3.9%. To calculate the strain transfer rate, graphene was initially relaxed on the copper slab. A uniaxial tensile strain of 1% was then applied only along the *x*-axis to the copper atoms and the overall lattice. During relaxation, atoms were allowed to move freely in-plane, while motion along the *z*-axis was constrained. The graphene–Cu surface distance was gradually increased using the same scheme to analyze its influence on interfacial adhesion and strain transfer between the copper substrate and graphene coating (Fig. 2b and c).

The calculated lattice parameters were 3.53 Å for copper and 2.46 Å for graphene. These values agree well with previous theoretical studies (3.52 Å for Cu and 2.44 Å for graphene) and experimental results (3.59 Å for Cu and 2.46 Å for graphene).^34^ Graphene can adopt three high-symmetry positions on the Cu(111) surface:^35^ top-fcc, top-hcp, and hcp–fcc. Among these, the top-fcc configuration was identified as the most stable and thus used in our calculations. After structural relaxation, the graphene–Cu distance was found to be 3.38 Å, similar to previously reported values (∼3.40 Å).^36^ The adhesion energy (*E*_adhesion_) was calculated using the following equation1
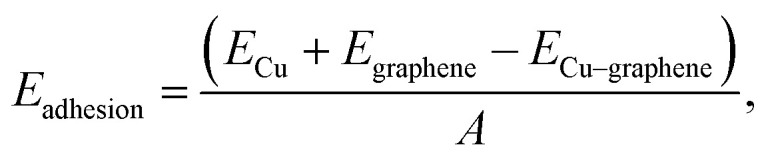
where *E*_Cu_ and *E*_graphene_ represent the total energies of isolated Cu(111) and graphene slabs, respectively, *E*_Cu–graphene_ is the energy of the combined slab system, and *A* is the interfacial area. At the relaxed geometry without strain, the calculated adhesion energy is 380 mJ m^−2^. This result is comparable to previously reported theoretical values of 394 mJ m^−2^ (using PAW and optB88-vdW functional)^37^ and 397 mJ m^−2^ (LDA).^38^ For comparison, the adhesion energy of monolayer graphene on a SiO_2_ substrate is 450 mJ m^−2^,^39^ which is not far away from that of graphene on Cu. However, the experimentally measured adhesion energy (740–1530 mJ m^−2^) is significantly higher.^40^ This discrepancy likely arises from a combination of experimental conditions such as organic contamination during surface preparation, defects in graphene sheets, copper grain boundaries, and/or surface roughness^41^ and can be seen in the experimental variation of graphene on various substrates.^39,42^

## Results and discussion

Graphene has two main Raman-active peaks: the G peak occurring at approximately 1580 cm^−1^ and the 2D peak occurring at approximately 2700 cm^−1^.^43^ In the case of the 2D peak, the intensity and line shape are dependent on the number of graphene layers up to bulk graphite,^43^ while the position of the peak depends on both the strain and carrier concentration.^44,45^ When uniaxial strain is applied, both the G peak and the 2D peak experience shifts in position and peak splitting due to the degeneracy lifting caused by the Poisson effect.^12^ Mohiuddin *et al.*^12^ reported a 2D peak shift of approximately 10–15 cm^−1^ when 0.11% uniaxial tensile strain is applied to monolayer graphene mechanically cleaved from bulk graphite *via* two-and four-point bending moments, and an approximately 50 cm^−1^ shift when 0.77% strain is applied. Because of the larger signal-to-noise ratio of the 2D peak in monolayer graphene on copper, its relatively large sensitivity to strain,^12^ and its relatively lower sensitivity to changes in doping,^44,45^ the 2D peak was chosen for the characterization studies reported here.

**Fig. 3:**
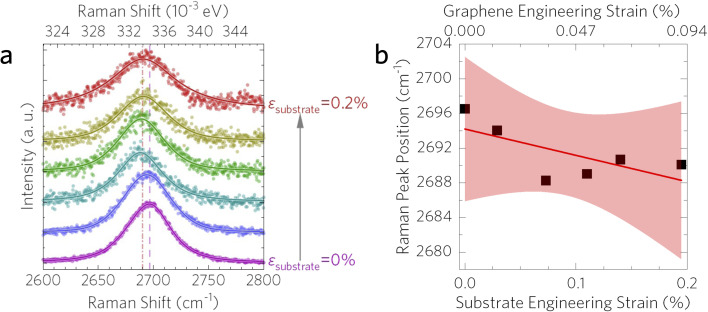
(a) Raman spectra and (b) peak position showing a red shift of the graphene 2D peak with respect to substrate engineering tensile strain excited at *P* = 2.9 mW. The shaded area in (b) indicates the 99% confidence interval band of a linear fit to the data.

Due to the graphene being measured on a Cu substrate, the Raman signal was not as intense as for suspended graphene or supported graphene on a SiO_2_-on-Si substrate.^46^ Although the experimental configuration chosen here yields a poorer signal, it is highly relevant to coating technologies used in applications where non-contact diagnostics are beneficial. Copper affects the Raman spectra by adding a strong background attributed to the surface plasmon emission of Cu^47^ and reducing the signal from a sharp and high-intensity 2D Raman peak to a broader and less intense peak. Fig. 3 shows the shift in Raman 2D peak position due to strain, along with the best-fit lines for each peak fit at each strain value. The strain induced in the graphene by strain applied to the copper substrate was calculated based on the peak shifts reported by Mohiuddin *et al.*^12^ as the ideal case (−64 cm^−1^/%, which assumes 100% strain transfer) in comparison with our experiment of graphene transferred onto Cu and annealed at 475 K (−32 cm^−1^/%), which indicated approximately 47% of strain was transferred to the graphene. This value was calculated using the lowest laser power such that the laser heating would not factor into the strain transfer rate. As our maximum substrate engineering strain was kept below 1%, the low strain transferred to the graphene meant that no peak-splitting was observed for our experiments. The strain transfer rate from the Cu substrate to the unannealed graphene was approximately 50 times lower than what we observed for the annealed graphene coating (0.9%, Fig. S3†).

In order to account for the strain induced by thermal expansion in graphene supported on a Cu substrate during heating experiments, the thermal expansion coefficients for Cu and graphene were calculated. Graphene is known to have a negative thermal expansion coefficient in temperatures ranging from 0–700 K.^48^ The graphene thermal expansion coefficient, *α*_graphene_, was obtained from the first-principles calculations of Mounet *et al.*^49^ from 0–500 K, and was fit with a polynomial equation as follows:

2 *α*_graphene_ × 10^6^ = −0.135371 − 0.319182*T* + 8.93359 × 10^−4^*T*^2^ − 7.61506 × 10^−8^*T*^3^,

where *T* is the sample temperature in Kelvin. For the case of Cu, the thermal expansion coefficient has been well-studied, and for the range of temperatures at which the Raman experiments were performed, it can be expressed as follows:^50^

3 *α*_Cu_ × 10^6^ = 11.504611 + 2.4346346 × 10^−2^*T* − 2.8812984 × 10^−5^*T*^2^ + 1.4737859 × 10^−8^*T*^3^.

The thermal mismatch strain between the graphene and the Cu substrate can be calculated as follows:^51^4
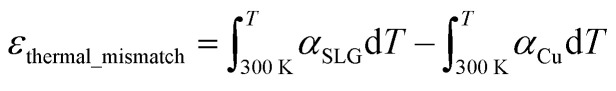


From this method, the strain mismatch for SLG on Cu is calculated to be −0.37% at 475 K; the Raman measurements in our experiment were taken at the same locations across all strains, laser powers, and ambient temperatures so that the extracted temperatures accounted for this effect. This thermal strain expansion may account for differences in Raman shift from sample to sample with heating and should be calibrated for each measurement location.

**Fig. 4:**
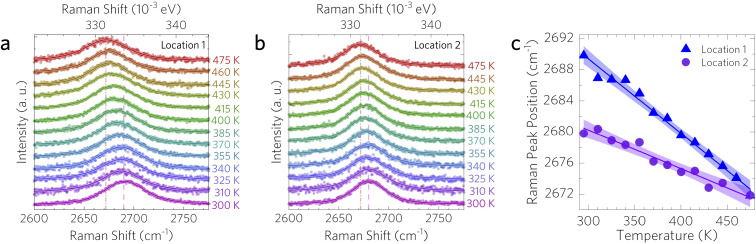
Temperature dependent Raman spectra for graphene on two different annealed copper coatings, denoted as (a) location 1 and (b) location 2. Spectra were taken under vacuum starting at the annealing temperature of 475 K and cooled to room temperature. (c) Graphene 2D peak position (blue triangles for location 1 and purple circles for location 2) showing the 99% confidence interval bands of linear fits to the data.

For the temperature dependence measurements at each strain, only the 2D peak was recorded to prevent grating and sample location drift. The Raman peak positions with respect to temperature were recorded in order to extract the *χ*_*T*_ coefficients needed to calculate the thermal conductivity and interfacial thermal conductance. The temperature dependence of the 2D peak in two graphene-coated copper samples is shown in Fig. 4. The shifts in 2D peak position from 475 to 300 K at two different coating locations were 27 cm^−1^ and 7.9 cm^−1^, with *χ*_*T*_ values of −0.096 ± 0.011 cm^−1^ K^−1^ and −0.050 ± 0.011 cm^−1^ K^−1^, respectively. The difference in slopes of the Raman peak temperature dependence may arise due to differences in graphene doping, pre-strain in the graphene and Cu substrate, and similar sample-to-sample variations. In the case of location 1, the initial Raman peak at 300 K starts at 2689.8 cm^−1^ and shifts to 2671.8 cm^−1^ at 475 K, while for location 2, the initial peak position at 300 K begins at 2679.8 cm^−1^ and shifts to 2671.8 cm^−1^ at 475 K.

**Fig. 5:**
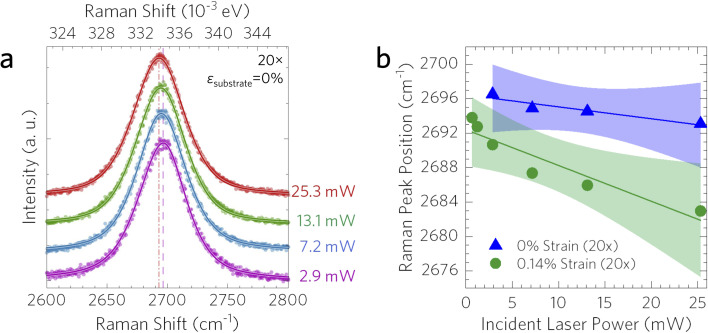
Power dependent Raman spectra for graphene on copper (method 1). (a) Incident laser power dependent (*P* = 2.9–25.3 mW) Raman spectra for unstrained graphene on copper, and (b) 2D peak position for 0% and 0.14% substrate engineering strain. Linear fits to the data are shown along with the 99% confidence interval bands.

Similar to the temperature dependence, the power dependence measurements were also only recorded in one spectral window centered on the 2D graphene peak in order to reduce uncertainty induced by small changes in grating position. At each strain value, all Raman spectra with respect to incident laser power were recorded within 9 minutes to minimize problematic sample *x*–*y*–*z* drift. Fig. 5 presents the laser power dependence of the 2D peak position at 0% strain as well as the linear fit obtained in order to extract *χ*_P_ for both the 0% strain and the 0.14% strain measurements for the one-objective method (sample 1). The *χ*_P_ values for 0% and 0.14% substrate engineering strain were −0.138 ± 0.032 cm^−1^ mW^−1^ and −0.416 ± 0.075 cm^−1^ mW^−1^, respectively, demonstrating that strain plays a significant role in the power dependent behavior of the Raman peak position shift. The uncertainty of these values was calculated from the standard error of all the data points present.

**Fig. 6:**
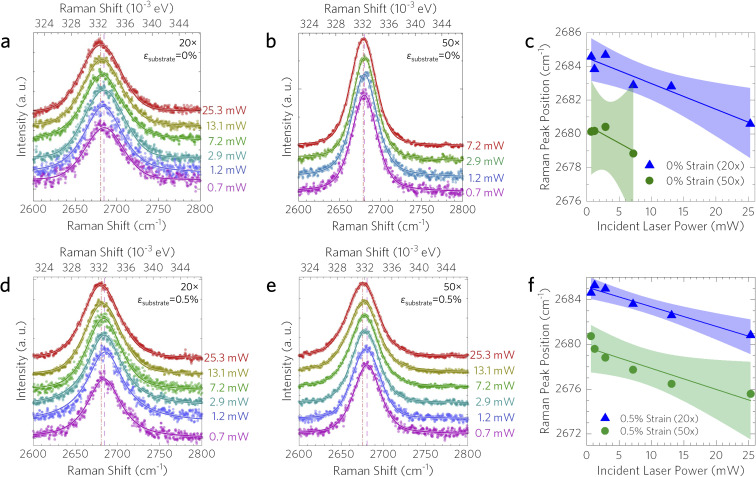
Power dependent Raman spectra for graphene on copper (method 2). Incident laser power dependent (*P* = 0.7–25.3 mW) Raman spectra taken using (a) 20× and (b) 50× objectives. (c) 2D peak position for 0% substrate engineering strain using 20× (blue triangles) and 50× (green circles) objectives. (d and e) Power dependent Raman spectra and (f) peak positions at 0.5% substrate engineering strain. Linear fits to the data are shown along with the 99% confidence interval bands.

To solve for the interfacial thermal conductance between the graphene coating and the Cu substrate, the heating profile of the laser can be varied by changing the objective lens; 50× and 20× objectives were used here. This was carried out only for the power dependence measurements, as the temperature dependence measurements did not need to be varied. The coating used in the two-objective method (method 2) had a strain transfer rate of 3.1%, lower than the one-objective coating (method 1), in addition to its lower *χ*_*T*_, indicating that it should have lower interfacial interaction. Fig. 6 presents the power dependence of the 2D peak in sample 2 for both 20× and 50× objectives at both 0% and 0.5% substrate engineering strain. The obtained values for *χ*_P_ at 0% applied strain were −0.151 ± 0.019 and −0.212 ± 0.082 cm^−1^ mW^−1^ for the 20× and 50× objectives, respectively; while the values for *χ*_P_ at the maximum 0.5% substrate engineering strain were −0.202 ± 0.038 and −0.302 ± 0.056 cm^−1^ mW^−1^ for the 20× and 50× objectives, respectively.

**Fig. 7:**
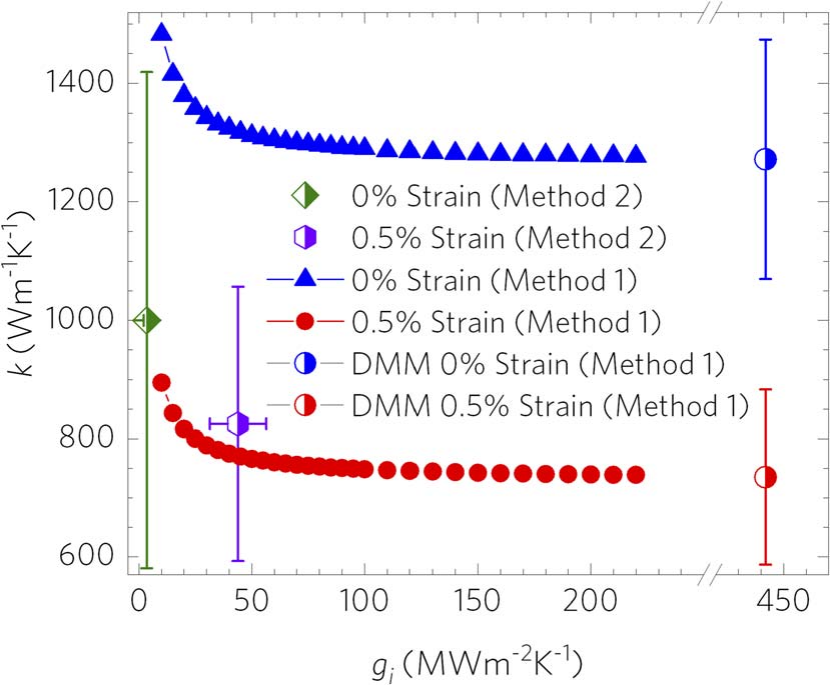
Relationship between interfacial thermal conductance (*g*_i_) and in-plane thermal conductivity (*k*) for monolayer graphene coated onto a Cu substrate. Analysis was performed using both one- and two-objective optothermal Raman thermometry techniques, denoted as methods 1 and 2, respectively.

By obtaining *χ*_P_ and *χ*_*T*_, the relevant thermal properties and uncertainty were extracted (see the ESI†). The *k* and total interface thermal conductance, *R*′′^−1^, which includes both *g*_i_ and substrate conductance in series, where 
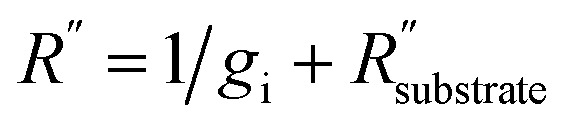
 and 1/*g*_i_ accounts for 40% of *R*′′ for the unstrained case and 5% of *R*′′ for the 0.5% strain case. For method 2, the values of *k* and *R*′′^−1^ were *k*_*ε*=0_ = 1000 ± 419 W m^−1^ K^−1^ and 
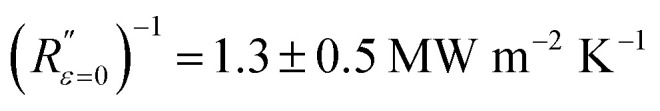
 at zero applied strain. At 0.5% substrate engineering strain, *k* decreased to *k*_*ε*=0.005_ = 825 ± 232 W m^−1^ K^−1^ and 
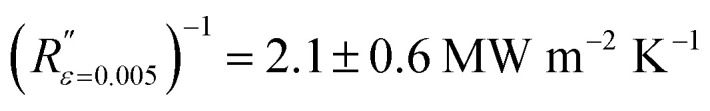
. From the relationship between the substrate resistance, interfacial conductance, and total contact resistance, the values for *g*_i_ work out to be *g*_i_ = 3.4 ± 1.3 MW m^−2^ K^−1^ for the 0% strain case and *g*_i_ = 43.9 ± 12.5 MW m^−2^ K^−1^. These results presented in Fig. 7 suggest that not only is the thermal conductivity subject to change due to strain, but that interfacial conductance, and therefore total contact resistance, will also change under strain; therefore, a multi-objective characterization method^8,23^ is useful to obtain a full picture of changes in thermal properties of coatings under strain.

For method 1, we note that the DMM *versus* experimentally reported *g*_i_ can differ by as much as an order of magnitude,^52^ and that it is well understood that the term *g*_i_ plays an important role in the final thermal conductivity extracted from laser heating experiments. The current experimental methods of extracting the interfacial conductance of graphene yield a value for *g*_i_ in the range of 10–100 MW m^−2^ K^−1^, according to thermal conductivity experiments conducted on monolayer graphene on various substrates.^8,53–57^ An example of the trend for how *g*_i_ affects the final value for *k* in the cases of both high and low strain is given in Fig. 7.

The coating used in method 1 was found to have an order of magnitude higher strain transfer rate than in method 2 and so we can expect a higher interfacial interaction strength, which justifies our use of the diffuse mismatch model (DMM) for *g*_i_ (442 MW m^−2^ K^−1^, the ideal interface assumption) (see the ESI†). Using this *g*_i_ value, we found *k*_*ε*=0_ = 4446 ± 1146 W m^−1^ K^−1^ to be approximately 4–6 times higher than that from method 2 (which was re-analyzed using method 1 for fair comparison). This value decreased continuously with increasing strain up to 0.14%, where *k* was 7 times lower (*k*_*ε*=0.0014_ = 642 ± 136 W m^−1^ K^−1^). The thermal conductivity rebounded to a value close to the unstrained *k* at higher strain, *k*_*ε*=0.00195_ = 4685 ± 937 W m^−1^ K^−1^. Potential factors for this include thin film relaxation and residual stress from transfer, comparable to effects seen by Mohiuddin *et al.*^12^ Additionally, any creep from the substrate and surface roughness may play a factor in these observed thermal conductivity shifts. More information on the thermal conductivity comparisons between methods 1 and 2 can be found in Fig. S4.†

To understand the non-linear strain dependence of the thermal properties and the low interfacial thermal conductance, the surface roughness of the substrate and coating was acquired by atomic force microscopy (AFM, Bruker Dimension Icon, Fig. S5†). When comparing the surface roughness values *r*_q_ and *r*_a_, the graphene-coated copper exhibited lower roughness (*r*_q_ = 7.8 ± 1.59 nm, *r*_a_ = 6.1 ± 1.11 nm) than the bare copper surface (*r*_q_ = 11.9 ± 4.41 nm, *r*_a_ = 9.0 ± 2.78 nm). This suggests that the graphene coating reduces the surface roughness by bridging the irregularities of the surface on which it is transferred. When comparing the *r*_q_ and *r*_a_ ratios, Cu had significantly larger surface irregularities (Cu *r*_q_/*r*_a_ = 1.62 ± 1.00), which were reduced after the graphene layer was applied (graphene-coated Cu *r*_q_/*r*_a_ = 1.37 ± 0.51), providing evidence that the graphene spanned across the peaks of the irregularities even after the annealing process. Therefore, it can be inferred that the graphene is freestanding over the grooves of the surface, where the convoluted effects of strain, phonon scattering, and contact area all play a role in the local thermal conduction properties. The interfacial thermal conductance is an order of magnitude lower than the ideal interface case (DMM), and thus, the AFM analysis gives us confidence that this is due to a low effective contact area. While this may not impact its performance as a barrier coating, the thermal properties will be a strong function of these nanometer-scale features. We note that the importance of post-assembly annealing to improve interfacial quality and thus interfacial thermal conductance has been demonstrated for graphene,^58,59^ transition metal dichalcogenide,^52^ and fully encapsulated transition metal dichalcogenide interfaces.^23^

Lastly, we note the strong dependence of interfacial configuration on interfacial properties, namely the adhesion energy and strain transfer calculated here by DFT, as the likely mechanism behind the significantly increased strain transfer, thermal conductivity, and strain dependence of thermal conductivity after annealing. Fig. 2 illustrates the strain transfer from the copper surface [Cu(111)] to graphene. Initially, graphene was relaxed on the Cu surface to reach an optimal interfacial distance. Then, a fixed tensile strain was applied along the *x*-axis to the copper lattice by adjusting only the positions of copper atoms. Following this, the graphene–copper interfacial distance was gradually increased from the relaxed equilibrium position, allowing atomic relaxation only within the plane (in-plane motion) while constraining vertical (out-of-plane) movement. This controlled procedure enabled the evaluation of how strain transfer efficiency varies with interfacial spacing. Under these conditions, at the optimal Cu–graphene separation, the strain transfer efficiency is highest, reaching about 91%. As the interfacial spacing increases above this optimum, the strain transfer efficiency sharply declines, with further separation lowering to 42% at a height of 0.64 Å above the optimum. This observation clearly indicates that maintaining a close and well-relaxed contact between copper and graphene is crucial for achieving effective strain transfer. Consequently, experimental factors such as organic contamination or surface imperfections will significantly reduce strain transfer performance. These findings underscore the importance of interface quality control in strain-engineering applications and emphasize the potential discrepancies between ideal theoretical calculations and practical experimental conditions, which will also influence the thermal properties of the graphene coating.

## Conclusion

For cases of graphene coatings transferred and annealed on a copper substrate, strain effects result in a decreasing thermal conductivity and increasing interfacial thermal conductance with increasing uniaxial tensile strain. These complex mechanical–thermal interactions at both the micro- and meso-scales are relevant for driving performance in functional coating and sensing applications, where the interfacial properties are shown here to govern the thermal response under deformation. The development of new methods for applying graphene coatings with improved interfacial properties^60^ will likely enhance the strain effects reported here.

## Conflicts of interest

There are no conflicts to declare.

## Supplementary Material

NA-007-D5NA00088B-s001

## Data Availability

The data supporting this article have been included in the manuscript and as part of the ESI.†
