# Supplementary material for: Thermal conductivity of graphene coated copper under uniaxial tensile mechanical strain
Source: Nanoscale Adv. 2025 May 8;7(12):3655–63. doi: 10.1039/d5na00088b (PMC12097146; doi:10.1039/d5na00088b)
Supplement: NA-007-D5NA00088B-s001 [file NA-007-D5NA00088B-s001.pdf]

Supplementary Information (SI) for Nanoscale Advances.
This journal is © The Royal Society of Chemistry 2025

Electronic Supplementary Information (ESI) for:

Thermal Conductivity of Graphene Coated Copper Under Uniaxial Tensile Mechanical Strain

Micah P. Vallin[1,2], Rijan Karkee[1], Hisato Yamaguchi[3], Chanho Lee[1], Ramon M. Martinez[1], Saryu J. Fensin[1], Jun Beom Park[1], Hi Tin Vo[4], Richard Z. Zhang[2,*], and Michael T. Pettes[1,*]

[1] *Center for Integrated Nanotechnologies, Materials Physics and Applications Division, Los Alamos National Laboratory, Los Alamos, NM 87545 USA*

[2] *Department of Mechanical Engineering, University of North Texas, Denton, TX 76207 USA*

[3] *Applied Electrodynamics Group, Accelerator Operations and Technology Division, Los Alamos National Laboratory, Los Alamos, NM 87545 USA*

[4] *Materials Science in Radiation and Dynamics Extremes (MST-8), Materials Science and Technology Division, Los Alamos National Laboratory, Los Alamos, NM 87545 USA*

[*] Email: zihao.zhang@unt.edu, pettesmt@lanl.gov

I. Governing thermal transport model

The optothermal Raman technique was developed by Balandin *et al.*[S1] as a non-contact method for determining the thermal conductivity of 2D materials from their Raman peak shifts due to varying laser power and temperatures. The technique as developed by Balandin *et al.* was used for suspended samples of graphene over a trench cut into an Si/SiO_2 substrate;[S1] properties such as the laser profile or substrate effects were not considered. The technique and analytical model was later refined by Cai *et al.*[S2] in order to calculate the thermal conductivity for graphene samples that were suspended over the circular holes of a gold-coated silicon nitride (Au/SiN_x) transmission electron microscopy grid. Their analytical model accounted for the graphene-substrate interfacial thermal conductance (g_i) in the region where the graphene is supported by the Au/SiN_x substrate, assumed to be isothermal although the non-negligible thermal resistance of the

thin support will prevent its function as a perfect heat sink in practice. The laser spot profile was modeled as a Gaussian and factored into the volumetric heat generation term [$\dot{q}(r)$]. Additionally, although the model did not consider the effects of radiation or convection heat transfer; these effects become important for experiments performed in air where convection can become appreciable in heated samples[S3], or at temperature extremes where radiation effects cannot be ignored.

For the purposes of the experiments analyzed here, the governing equation used to obtain the graphene coating's temperature profile is similar to previous reports (e.g. refs. S3, S4), but also accounts for the effects of substrate resistance, convection, and radiation based on conservation of energy as[S5]

$$\frac{d^2T}{dr^2} + \frac{1}{r}\frac{dT}{dr} - \frac{1/R'' + h_{\mathrm{conv}}(T)}{kt_{\mathrm{sample}}}(T - T_\infty) - \frac{h_{\mathrm{rad}}(T)}{kt_{\mathrm{sample}}}(T - T_{\mathrm{surr}}) + \frac{\dot{q}_{\mathrm{gen}}(r)}{k} = 0, \qquad \text{(S1)}$$

where T_∞ is the ambient temperature of the convective medium and the substrate, and T_{surr} is the temperature of the surrounding environment radiatively exchanging energy with the sample. For the purpose of our room temperature experiments, both T_∞ and T_{surr} are 300 K. The R'' term combines both the interfacial conductance (g_i) and the shape factor for the substrate resistance effects, approximated as $R''_{\mathrm{substrate}} = t_{\mathrm{substrate}}/k_{\mathrm{substrate}}$. The substrate thickness was measured using a Keyence VHX-7000 calibrated digital microscope as $t_{\mathrm{substrate}}$=180 μm. The shape factor for the substrate resistance was assumed to be a plane wall due to the heat dissipating down from the laser in the z-direction being assumed to be greater than heat dissipating along the r-direction within the substrate. The value for $k_{\mathrm{substrate}}$ was taken as k_{Cu} = 401 $\mathrm{Wm^{-1}K^{-1}}$.[S6] The temperature-dependent convection coefficient, h_{conv} was determined by the Nusselt number correlation for free convection from the upper surface of a hot plate ($Nu_L = 0.54 Ra_L^{1/4}$), where L is a characteristic length

determined by the sample surface area divided by the perimeter of the sample, and Ra_L is the Rayleigh number across this length), $h_{\mathrm{rad}}(T) = \varepsilon\,\sigma\,(T^2+T_{\mathrm{surr}}^2)\,(T+T_{\mathrm{surr}})$, and $\dot{q}_{\mathrm{gen}}(r)$ is the laser power absorbed per unit volume assuming a Gaussian profile for the laser beam spot

$$\dot{q}_{\mathrm{gen}}(r) = \frac{2P\alpha}{\pi r_0^2 t_{\mathrm{sample}}} \exp\left(-\frac{2r^2}{r_0^2}\right), \tag{S2}$$

where P is the incident laser power and α is the sample absorption, and $r_0 = 2\cdot\sigma$ where σ is the variance. The incident power $P = \int P_0''\cdot\exp(-2r^2/r_0^2)\cdot r dr d\phi$, thus $P_0'' = 2P/(\pi r_0^2)$, and we define $r_0 \equiv \lambda/(n\cdot\pi\cdot\mathrm{NA})$ as the beam radius at focus,[S7] where n is the refractive index of air, λ is the wavelength of the laser, and NA is the numerical aperture of the objective lens.

Calculating the absorption of the graphene sample on the Cu substrate poses its own set of challenges and is one of the most critical values needed to quantify thermal conductivity from laser-based metrology techniques. Overestimating this value also overestimates the extracted thermal conductivity.

One possible direct way to calculate the absorption coefficient of graphene on the Cu substrate is to measure the laser line intensity on and off the graphene sample to calculate the reflectivity ($\rho = I_{\mathrm{laser,\ sample}} / I_{\mathrm{laser,\ substrate}}$), assuming no transmissivity occurs, and using the equation $\alpha = 1 - \rho$. However, factors such as impurities in the sample, pre-strain, and the thermalization from laser heating can affect the reflectivity and may therefore not be a reliable method to calculate absorption.

Thus, in our analyses, we instead calculate the radiative properties of graphene on the Cu substrate using the transfer matrix method (TMM)[S8, S9] to obtain the absorptance, reflectance, and transmittance of the 2D coating. Since the incident angle of the laser is normal, transverse electric and transverse magnetic polarizations yield the same radiative properties. We obtained the

refractive indices of graphene at wavelengths from 200 nm to 1.0 μm from Weber *et al.*,[S10] and indices of Cu from Babar & Weaver.[S11] The TMM assumes that the materials in the multilayer structure are homogeneous and isotropic; the graphene can be assumed to be isotropic due to only considering the in-plane properties during Raman scattering. The matrices which compose the TMM are the propagation matrix and the dynamical matrix for each material, which can be described as

$$P_1 = \begin{bmatrix} 1 & 0 \\ 0 & 1 \end{bmatrix}, \tag{S3}$$

$$P_l = \begin{bmatrix} \exp\left(-ik_{z,l}d_l\right) & 0 \\ 0 & \exp\left(ik_{z,l}d_l\right) \end{bmatrix} \text{ for } l \geq 2, \text{ and} \tag{S4}$$

$$D_l = \begin{bmatrix} 1 & 1 \\ \frac{k_{z,l}}{\varepsilon_l} & -\frac{k_{z,l}}{\varepsilon_l} \end{bmatrix}, \tag{S5}$$

where d_l and $k_{z,l}$ represent the thickness and wave vector in the l-th layer of the multilayer structure respectively, and ε_l represents the l-th layer's dielectric function. In this study the layers are graphene and copper. The propagation matrix and dynamical matrices are used to obtain the matrix product, M, as

$$M = \begin{bmatrix} M_{11} & M_{12} \\ M_{21} & M_{22} \end{bmatrix} = \prod_1^{N-1} P_l\, D_l^{-1} D_{l+1}\,, \tag{S6}$$

where the reflectance and transmittance properties, R_λ, and T_λ, respectively, can be obtained as

$$R_\lambda = \left|\frac{M_{21}}{M_{11}}\right|^2, \text{ and} \tag{S7}$$

$$T_\lambda = \frac{\mathrm{Re}(k_{Nz}/\varepsilon_N)}{\mathrm{Re}(k_{1z}/\varepsilon_1)} \left|\frac{1}{M_{11}}\right|^2. \tag{S8}$$

In equation S6, N represents the total number of layers in the multilayer structure and from equations S6–S8, the absorptance can be calculated as $A_\lambda = 1 - R_\lambda - T_\lambda$. Using the assumption that the thickness of the monolayer graphene is half the c-axis lattice parameter of highly ordered graphite, 0.335 nm,[S12] the absorptance value of the graphene on the Cu substrate, which is used as the value of α in equation S2, is $A_\lambda = \alpha = 0.0106$, which is 46% of the value usually recorded for monolayer graphene (α = 0.023)[S13] and hence in our model we may underestimate the graphene thermal conductivity by approximately a factor of two. We note the importance of the value of α taken when extracting thermal conductivity from laser-based methods and ask the reader to note this value when comparing different reports on graphene thermal conductivity.

II. Analytical extraction of thermal properties

From equation S1, the only undefined variables that affect the temperature profile are the graphene-substrate interfacial thermal conductance and graphene thermal conductivity, g_i and k, respectively. This work presents two cases for obtaining the values for k and g_i. The first method is where g_i is calculated separately from k where only one power dependent Raman measurement is needed (one-objective method, denoted method 1); g_i was calculated here using the Diffuse Mismatch Model (DMM)[S14] based on the phonon group velocities obtained from literature of graphene[S15] and Cu[S16]; this method is further outlined in literature as a way to calculate g_i between two interfaces and can incorporate vibrational density of states as a factor as well.[S9, S14, S16] The calculated value of g_i using the DMM is $g_i = 4.42\times10^8$ $Wm^{-2}K^{-1}$. Using this value for g_i, the extraction of k can be performed as a back calculation using the temperature measured within the laser spot radius, T_m. The second method for calculating the values for k and g_i is to vary the laser heating profile. This is typically performed by taking power dependent Raman measurements with

two different microscope objectives (two-objective method, denoted method 2). To approximate this temperature using equation S2 for the laser heating profile, a weighted mean temperature, T_m, is defined as

$$T_m = \frac{\int_0^\infty rT(r)\dot{q}(r)dr}{\int_0^\infty r\dot{q}(r)dr} \,. \tag{S9}$$

Equation S9 yields a numerical solution which converges once $\partial T_m/\partial P = \chi_P/\chi_T$, where χ_P is the coefficient of Raman shift with respect to laser power (in units of cm^{-1}W^{-1}) and χ_T represents the coefficient of Raman shift with respect to temperature (in units of cm^{-1}K^{-1}). In the case of the two-objective method, the numerical solution is said to have converged once the ratio of $\partial T_m/\partial P$ values for both objectives equal the ratio of the χ_P/χ_T values. In this work, the higher signal-to-noise ratio of the 2D peak along with the 12-minute reduction in acquisition time gained by solely measuring the 2D peak as opposed to the full spectra is why we have ultimately decided to focus on the Raman shift of the 2D peak for the strained graphene coating. We note that in principle any Raman peak can be used for this analysis as long as it is calibrated by the χ_T measurement.

The uncertainty in k can be quantified using the uncertainty propagation formula considering uncertainties in sample thickness, power and temperature dependent Raman shifts, and absorption coefficient as

$$\frac{u_k}{k} = \sqrt{\left(\frac{u_{t_{\text{sample}}}}{t_{\text{sample}}}\right)^2 + \left(\frac{u_{\chi_T}}{\chi_T}\right)^2 + \left(\frac{u_{\chi_P}}{\chi_P}\right)^2 + \left(\frac{u_\alpha}{\alpha}\right)^2} \,. \tag{S10a}$$

where u denotes the uncertainty of the parameter in the subscript. Likewise, the uncertainty values for g_i can be defined as

$$\frac{u_{g_i}}{g_i} = \sqrt{\left(\frac{u_{t_{\text{sample}}}}{t_{\text{sample}}}\right)^2 + \left(\frac{u_{\chi_T}}{\chi_T}\right)^2 + \left(\frac{u_{\chi_P}}{\chi_P}\right)^2 + \left(\frac{u_\alpha}{\alpha}\right)^2} \,. \tag{S10b}$$

III. Additional experimental results

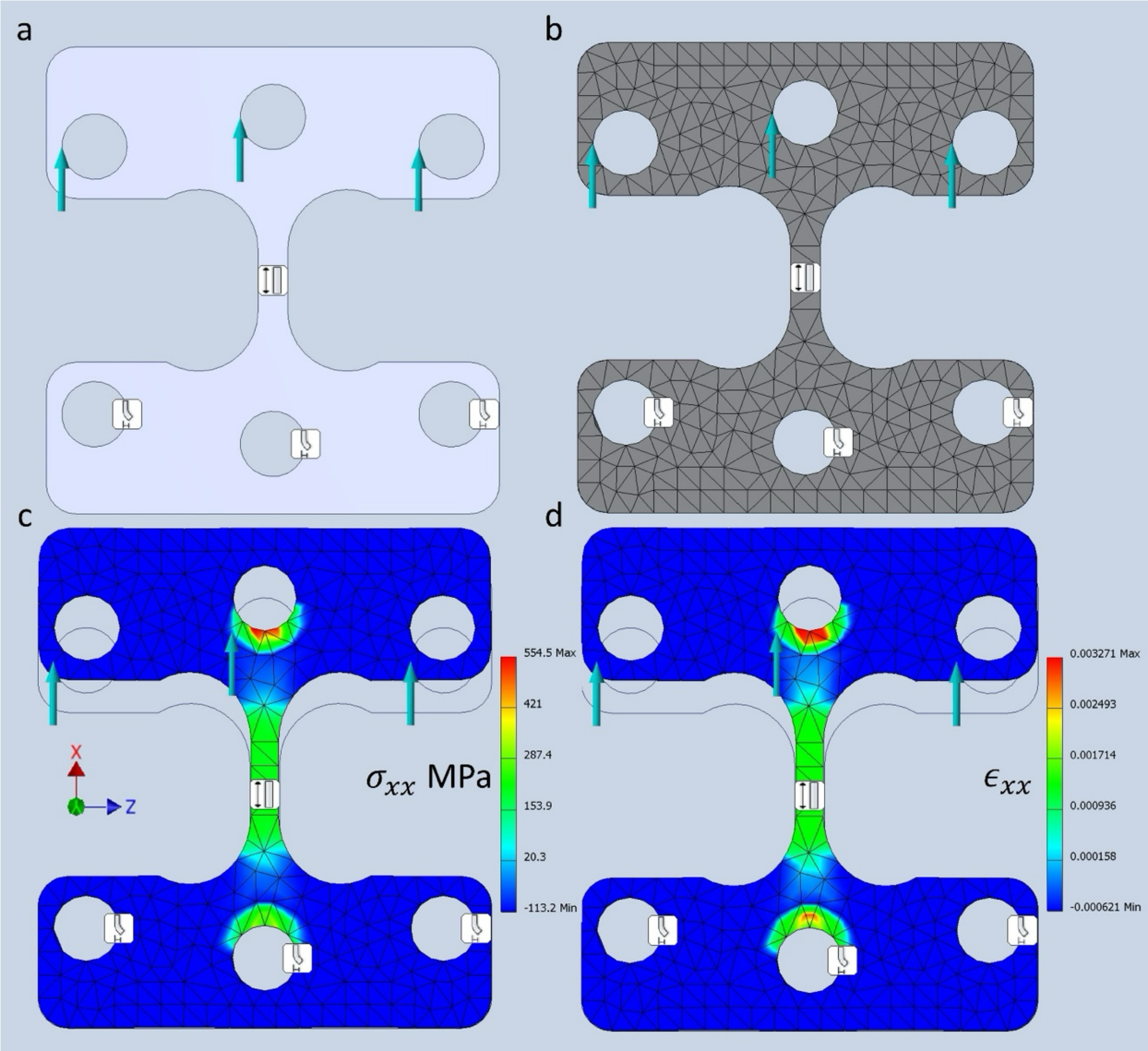

Fig. S1. To confirm test geometry would not lead to stress concentration at the shoulders, a simple static stress analysis was performed on the geometry using Inventor Professional 2023. (a) Geometry of the tensile specimen (top view). (b) Meshing used for the static analysis. Fixed constraints were placed on the one end of the tensile bar (lower end). For the tensile simulation, we prescribe a 100 μm displacement on the three holes on the other end of the tensile bar (top end). (c) Normal stress distribution along the loading direction. The stress is concentrated at the gauge section. There is no stress concentration near the shoulders. Note that there is some degree of stress concentration near the holes where displacement is applied. However, in the experiment, we secured the cross head with screws to prevent deformation near the holes. (d) Normal strain along the loading direction.

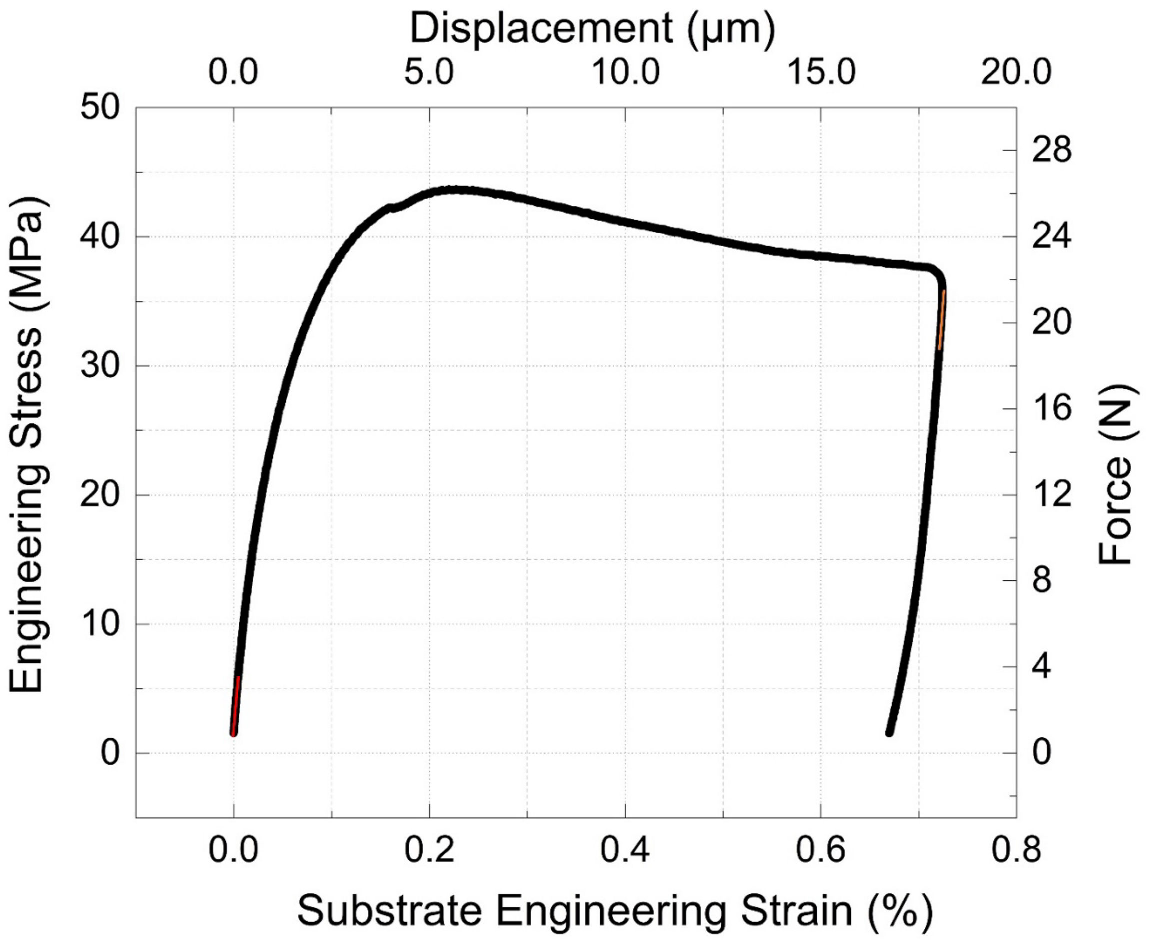

Fig. S2. Stress-strain curve taken to validate the engineering strain in the Cu substrate up to the plastic deformation regime. The strain cell was first pre-strained to its maximum travel before the specimen was mounted, ensuring the full displacement range during testing. Linear fits of the red- and orange-highlighted elastic regions give elastic moduli of 104.2 ± 2.0 GPa and 106.4 ± 2.9 GPa, respectively.

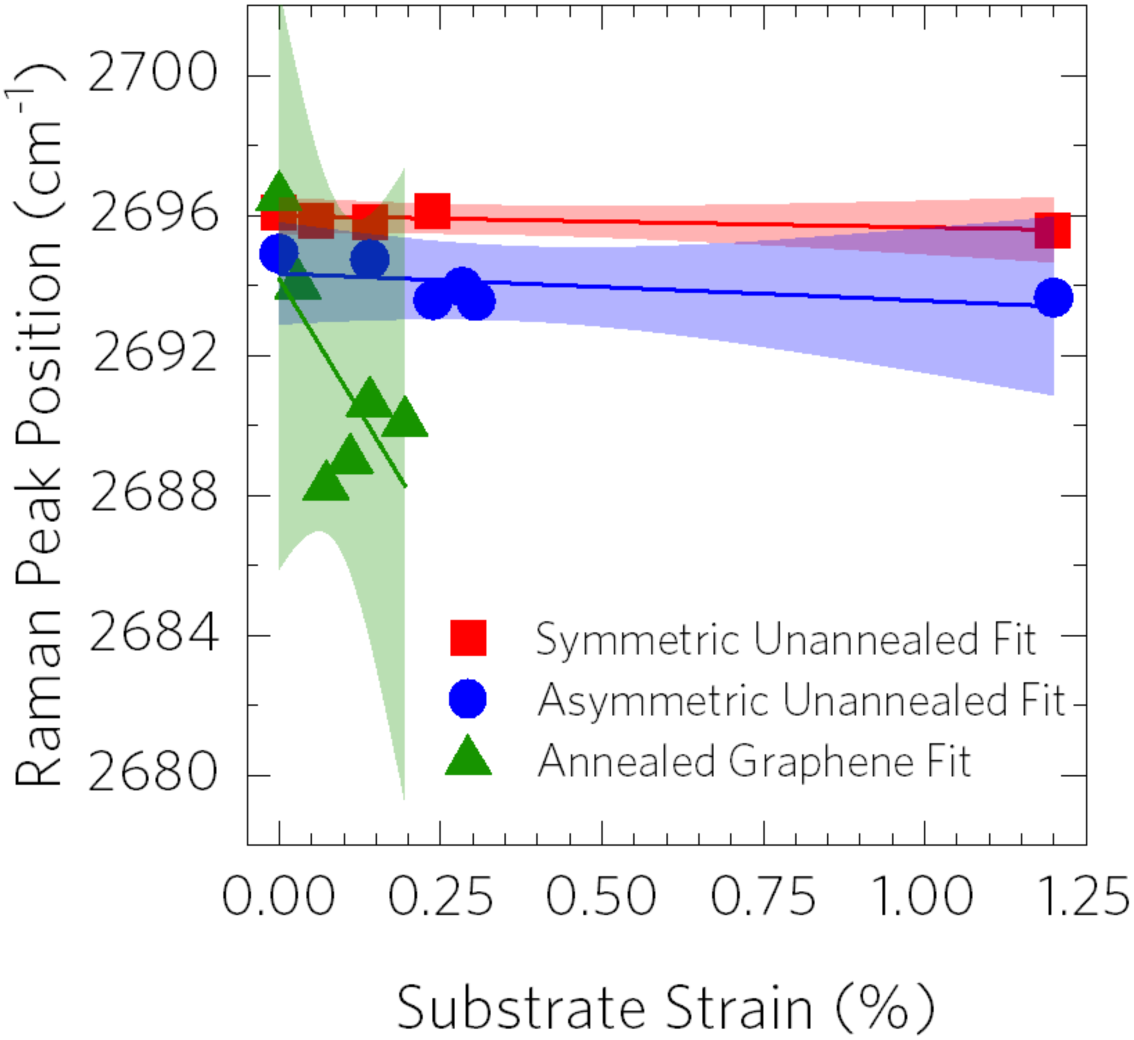

Fig. S3. Comparison of strain-induced Raman peak shifts in graphene transferred onto a polished Cu substrate. Unannealed coating [fit with a symmetric Gaussian-Lorentzian peak (red squares) and fit with an asymmetric Gaussian-Lorentzian peak (blue circles)] and annealed coating fit with a symmetric Gaussian-Lorentzian peak (green triangles)] are shown for comparison. The unannealed graphene yields a drastically lower strain transfer rate ($\varepsilon_{graphene}/\varepsilon_{substrate}$ = 0.9±0.3%) than the annealed case (47%).

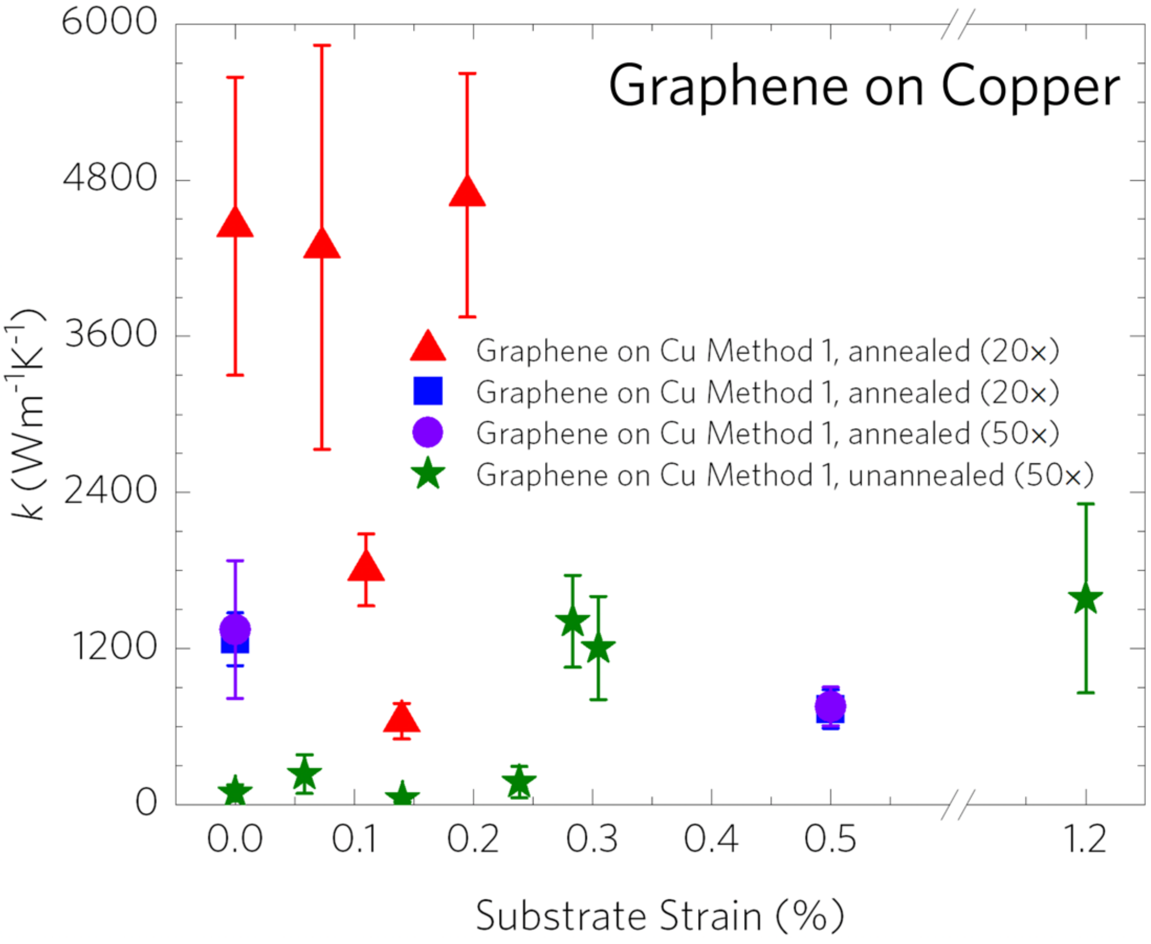

Fig. S4. Comparison of thermal conductivity values for the one-objective method analyzed according to method 1 with either only the 20× or only the 50× objective data. This analysis assumes an interfacial thermal conductance of g_i=4.42×10^{8} Wm^{-2}K^{-1} according to the diffuse mismatch model. The cases where the graphene was annealed after transfer to the copper substrate show a trend where the thermal conductivity decreases as substrate strain increases while the unannealed case shows a stepwise trend where thermal conductivity increases substantially only after 0.25% likely due to a change in g_i not captured by method 1. These results demonstrate that increasing the interfacial interaction by annealing the graphene coating after transfer to the substrate is important for increasing strain transfer, as can be expected.

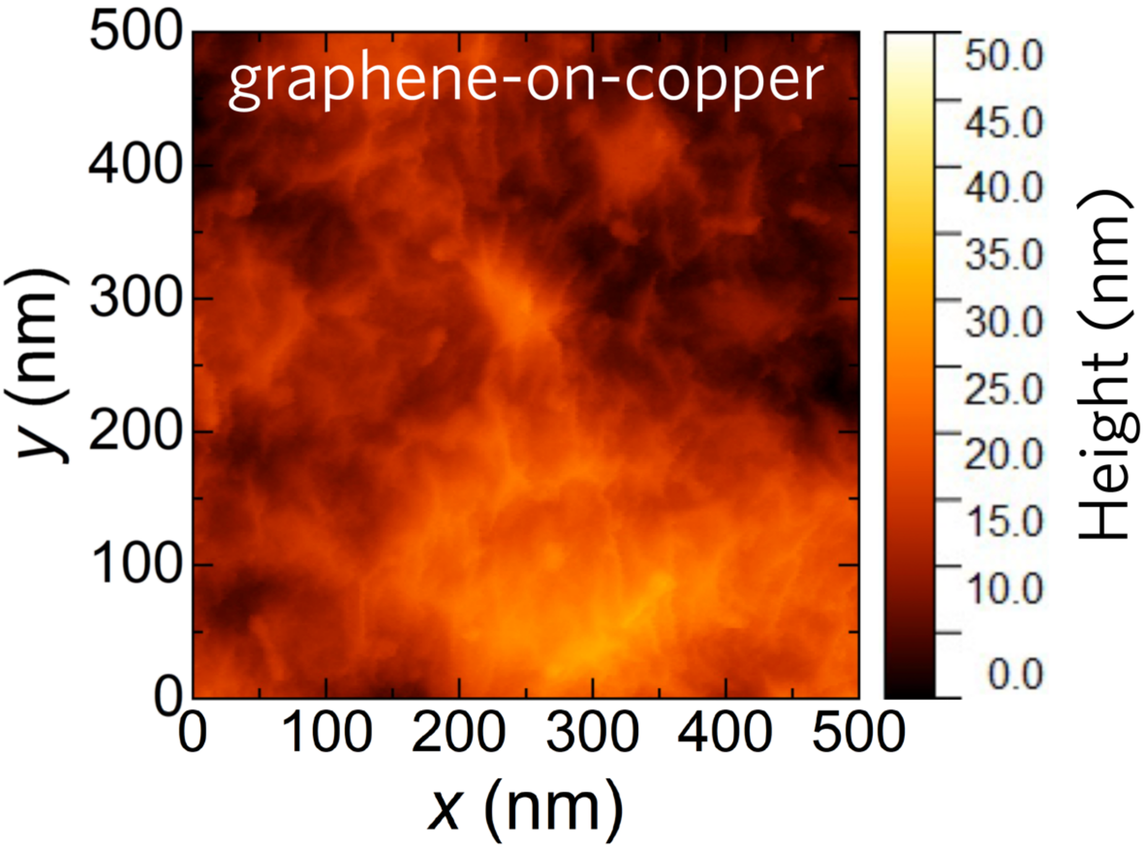

Fig. S5. Atomic force microscopy surface image of graphene coated onto copper after annealing at 475 K.

Supplementary references

S1. A. A. Balandin, S. Ghosh, W. Z. Bao, I. Calizo, D. Teweldebrhan, F. Miao and C. N. Lau, *Nano Lett.*, 2008, **8**, 902–907.

S2. W. Cai, A. L. Moore, Y. Zhu, X. Li, S. Chen, L. Shi and R. S. Ruoff, *Nano Lett.*, 2010, **10**, 1645–1651.

S3. I.-K. Hsu, M. T. Pettes, M. Aykol, L. Shi and S. B. Cronin, *J. Appl. Phys.*, 2010, **108**, 084307.

S4. Y. Wang and X. Zhang, *J. Appl. Phys.*, 2024, **136**, 074302.

S5. T. L. Bergman, A. S. Lavine, F. P. Incropera and D. P. DeWitt, *Fundamentals of Heat and Mass Transfer*, John Wiley & Sons, New York, 7th edn., 2011.

S6. Y. S. Touloukian, R. W. Powell, C. Y. Ho and P. G. Klemens, in *Thermophysical Properties of Matter*, eds. Y. S. Touloukian and C. Y. Ho, IFI/Plenum, New York, 1970, vol. 1.

S7. O. Svelto, *Principles of Lasers*, Springer-Verlag US, New York, 5th edn., 2010.

S8. R. Z. Zhang and Z. M. Zhang, *J. Quant. Spectro. Rad. Trans.*, 2015, **158**, 91–100.

S9. Z. M. Zhang, *Nano/Microscale Heat Transfer*, Springer Nature Switzerland AG, Cham, Switzerland, 2nd edn., 2020.

S10. J. W. Weber, V. E. Calado and M. C. M. van de Sanden, *Appl. Phys. Lett.*, 2010, **97**, 091904.

S11. S. Babar and J. H. Weaver, *Appl. Opt.*, 2015, **54**, 477–481.

S12. J. Y. Howe, C. J. Rawn, L. E. Jones and H. Ow, *Powder Diffr.*, 2003, **18**, 150–154.

S13. R. R. Nair, P. Blake, A. N. Grigorenko, K. S. Novoselov, T. J. Booth, T. Stauber, N. M. R. Peres and A. K. Geim, *Science*, 2008, **320**, 1308–1308.

S14. G. Chen, *Nanoscale Energy Transport and Conversion: A Parallel Treatment of Electrons, Molecules, Phonons, and Photons*, Oxford University Press, New York, 2005.

S15. X. Cong, Q.-Q. Li, X. Zhang, M.-L. Lin, J.-B. Wu, X.-L. Liu, P. Venezuela and P.-H. Tan, *Carbon*, 2019, **149**, 19–24.

S16. L. De Bellis, P. E. Phelan and R. S. Prasher, *J. Thermophys. Heat Transf.*, 2000, **14**, 144–150.
